# Story of Mrs. Siddons

**Published:** 1874-12

**Authors:** 


Story of Mrs. Siddons.—"When I was a poor girl," relates Mrs. Siddons, the actress, "working very hard for my thirty shillings a week, I went down to Liverpool during the holidays, where I was kindly received. I was to perform in a new piece, something like those pretty little affecting dramas they get up now at the minor theatres; in my character I represented a poor friendless orphan girl, reduced to the most wretched poverty. A heartless tradesman prosecutes the sad heroine for a heavy debt, and insists on putting her in prison unless some one will be bail for her. The girl replies, 'Then I have no hope—I have not a friend in the world.' 'What! will no one be bail for you to save you from prison?' asks the stern creditor. 'I have told you I have not a friend on earth,' was my reply. But just as I was uttering the words, I saw a sailor in the upper gallery springing over the railing, letting himself down from one tier to another, until he bounded clear over the orchestra and footlights, and placed himself beside me in a moment. 'Yes, you shall have one friend at least, my poor young woman!' said he, with the greatest expression in his honest, sunburnt countenance. "I will go bail for you to any amount! And as for you (turning to the frightened actor), if you don't bear a hand and shift your moorings, you lubber, it will be the worse for you when I come athwart your bows.' Every creature in the house rose; the uproar was perfectly indescribable—peals of laughter, screams of terror, cheers from the tawny messmates in the gallery, preparatory scraping of violins in the orchestra; and amid the universal din, there stood the unconscious cause of it sheltering me, 'the poor distressed young woman,' and breathing defiance and destruction against my mimic persecutor. He was only persuaded to relinquish his care of me by the manager's pretending to arrive and rescue me with a profusion of theatrical bank notes."

